# Functional Outcomes of Surgery in Cervical Spondylotic Radiculopathy versus Myelopathy: A Comparative Study

**DOI:** 10.1155/2013/293806

**Published:** 2013-08-18

**Authors:** F. Omidi-Kashani, E. G. Hasankhani, M. F. Vavsari, S. Afsari, F. Golhasani-Keshtan

**Affiliations:** ^1^Orthopedic Research Center, Orthopedic Department, Imam Reza Hospital, Mashhad University of Medical Sciences, Mashhad 9137814864, Iran; ^2^Orthopedic Department, Imam Reza Hospital, Mashhad University of Medical Sciences, Mashhad, Iran; ^3^Orthopedic Research Center, Ghaem Hospital, Mashhad University of Medical Sciences, Mashhad, Iran

## Abstract

*Background*. Cervical spondylosis can cause three different categories of symptoms and signs with possible overlap in the affected patients. *Aim*. We aim to compare functional outcome of surgery in the patients with cervical spondylotic radiculopathy and myelopathy, regardless of their surgical type and approach. *Materials and Methods*. We retrospectively reviewed 140 patients with cervical spondylotic radiculopathy and myelopathy who had been operated from August 2006 to January 2011, as Group A (68 cases) and Group B (72 cases), respectively. The mean age was 48.2 and 55.7 years, while the mean followup was 38.9 and 37.3 months, respectively. Functional outcome of the patients was assessed by neck disability index (NDI) and patient satisfaction with surgery. *Results*. Only in Group A, the longer delay caused a worse surgical outcome (NDI). In addition, in Group B, there was no significant relationship between imaging signal change of the spinal cord and our surgical outcomes. Improvement in NDI and final satisfaction rate in both groups are comparable. *Conclusions*. Surgery was associated with an improvement in NDI in both groups (*P* < 0.001). The functional results in both groups were similar and comparable, regarding this index and patient's satisfaction score.

## 1. Introduction

Spondylosis is the most common cause of neural compression in cervical spine [1]. The disease can cause three different categories of symptoms and signs with possible overlap in the affected patients [2, 3]. These patients may complain of the neck (pain, stiffness, and limited range of motion) or suffer from radiculopathy or even myelopathy [4]. Neurologic symptoms are usually aroused when the space available for the neural elements is reduced by osteophytes, hypertrophied ligamentum flavum, or a herniated disc [4]. In those patients whose main manifestation of the disease is neck complains, conservative treatment is usually recommended, while in some with cervical spondylotic radiculopathy (CSR) or myelopathy (CSM), surgery may be associated with better satisfactory outcomes [5–7]. Although, some authors still have doubts about the long-term results of surgery in these cases [2, 8].

The poor prognostic factors usually quoted in the surgical treatment of the patients include older age, abnormal cervical curvature, multisegmental compression, more duration of symptoms, higher number of comorbidities, decreased signal intensity on T1-weighted images, increased signal intensity on T2-weighted images, and existence of cord atrophy in preoperative magnetic resonance images (MRIs) [1, 5, 6, 9–11]. Although many papers have been published about the surgical outcome of the patients with CSM or CSR, according to our knowledge, very few studies have been conducted to compare the two. In this retrospective study, we aim to compare functional outcome of surgery in patients with CSR and CSM. 

## 2. Materials and Methods

 In this study, following local institutional review board approval (code no. 910106), we retrospectively reviewed our patients with CSM and CSR who had been operated from August 2006 to January 2011. We arbitrary placed our patients into two groups. Those patients with predominant manifestation of CSR were placed in Group A, while the others were placed in Group B. In rare cases, who complained of both conditions with equal intensity, due to the differences in the clinical significance, we conventionally put them in the second group. Our inclusion criteria included refractory complains to aggressive medical treatment more than six weeks, significant neurologic deficit (especially if progressive), and a followup period more than 24 months. We excluded those patients with secondary spondylosis (due to previous trauma, congenital anomalies, and infectious or inflammatory diseases), significant underlying disease (Uncontrolled diabetes mellitus, severe osteoporosis, etc.), tandem stenosis (significant accompanying spinal stenosis in another area of the spine), and those who had history of previous surgery.

 Preoperatively, in Group B, the presence of increased signal intensity inside the cord on T2-weighted MRI was assessed by two self-governing radiologists and was described as positive when they both were in concurrence. We did not grade this increased intensity and only classified it as normal or positive. In this study, surgical techniques and approaches were not considered as the effective variables, and we primarily aimed to efficiently decompress the neural elements and stabilize the spine if necessary. In our opinion, it matters little that this goal would be achieved with anterior or posterior approach, spondylodesis or disc arthroplasty, anterior cervical discectomy or corpectomy, and laminectomy or laminoplasty; but each technique or approach should be used in its proper patient. After the informed consent form was signed, demographic and imaging data were recorded, and then the patient operated. All the procedures have been carried out by the fist author (FOK) with a relative similar technique during this period of time.

Functional outcome of the patients was assessed by two questionnaires. We used neck disability index (NDI) as an assessment tool to evaluate functional outcome [12]. Translation and validation study of the Iranian version of this international questionnaire has been already performed by Mousavi in 2007 and proved to be extremely reliable [13]. The NDI questionnaire was completed preoperatively and then every six months after surgery. And patient satisfaction with surgery was also evaluated subjectively. At the last visit, the cases were asked to choose one of the following responses regarding their satisfaction with the surgical treatment, according to criteria adopted from the North American Spine Society Low Back Outcome Instrument: (1) surgery met my hopes, (2) I did not improve as much as I had hoped, but I would undergo the same surgery for the same outcome, (3) surgery helped, but I would not undergo the same treatment for the same outcome, or (4) I am the same as or worse than I was before the surgery [14]. 

### 2.1. Statistical Analysis

 We used independent samples *t*-test for comparison and Pearson and Kendall's Tau-b correlation coefficients for measuring the dependency. Statistical Package for the Social Sciences (SPSS) software version 11.5 was used for statistical analysis. *P* < 0.05 was considered significant statistically. 

## 3. Results

Initially, 150 patients were eligible for inclusion, but later ten patients due to short duration of followup were excluded. Eventually, the study was performed on 140 patients. Demographic data and the duration of followup are shown in Table 1. All the surgical approaches in Group A were carried out from anterior, while 19.5% of the procedures in Group B were performed posteriorly. We operated no patient with combined anterior and posterior approach. In 52 (76.5%) of the patients with radiculopathy (Group A), there was only one level of cervical involvement, and in the remaining 16 (23.5%), two levels of involvement were present. In contrary to Group A, in myelopathy group, only 22 (30.6%) of the patients had a monosegmental involvement, and two, three, four, and five levels of involvement were observed in 20 (27.8%), 16 (22.2%), 6 (8.3%), and 8 (11.1%), respectively. 

 Regarding to surgical delay (the time interval between appearances of complains and surgery) and its relation to functional recovery (NDI), based on Pearson's correlation coefficient, in Group A, the longer delay caused a worse surgical outcome (NDI), while this relationship was not observed in Group B. In addition, in Group B, there were 30 cases (41.7%) with increased signal intensity inside the cord preoperatively, and based on Kendall's Tau-b correlation coefficient, there was no significant relationship between this imaging signal change and our surgical outcomes (correlation coefficient of 0.12, *P* = 0.204 for final NDI). 

Functional status of the patients (NDI) before surgery and at last followup visit is shown in Table 2. Patient's satisfaction from the surgery was also assessed at the last followup and depicted in the same table. Although in comparing CSR with CSM patients, satisfactory rates from the surgery seemed to be higher in the first group, this was not significant, statistically. Patients' satisfaction scores were highly correlated with final NDI scores. Overall, surgery could significantly improve NDI in both groups (*P* < 0.001). Regarding to improved NDI and patient's satisfaction score, functional results in both groups were similar and comparable. 

 In this study, we had three implant failures (all in Group A; screw loosening), and three pseudarthroses (two case in Group A and one in Group B). Five cases needed reoperation (three due to symptomatic pseudarthrosis, one adjacent segment disease, and one symptomatic device loosening). One patient in Group A had a transient paralysis of the recurrent laryngeal nerve, lasting for two months. Three patients had superficial wound infection, all treated by local wound care and antibiotic therapy.

## 4. Discussion

 We investigated 140 patients with refractory cervical spondylosis who had been treated with surgery. Our study showed that the functional results of surgery and patients' satisfaction rate in CSR compared to CSM are apparently better but statistically comparable. 

Usually, it is said that CSM compared to CSR more commonly occurred in older patients [15, 16]. In this study, we were unable to find a significant difference in the age distribution. The sex ratio of the patients reported in different studies is highly variable [6, 11, 16–18]. The sex ratios of our patients in the two groups were markedly different from each other; radiculopathy was more common in women, whereas the opposite is myelopathy.

Several surgical prognostic factors have been reported in the literature. In a prospective study, Furlan and coauthors evaluated 81 patients with CSM who underwent decompressive surgery [17]. They finally showed that surgical treatment of CSM is associated with appropriate functional outcome. Older age and greater number of underlying preoperative comorbidities are associated with lower surgical outcome. Karpova et al. in another study on 65 patients with surgically treated CSM found that good prognostic factors in these cases included younger age and lower preoperative baseline modified Japanese Orthopaedic Association score, but the severity of cord compression, signal intensity change on magnetic resonance imaging scans, and treatment delay have little prognostic value [19]. Treatment delay was associated with lower preoperative functional status of the patients. Our research results also confirmed that signal change and treatment delay had no adverse effect on functional outcome of these patients. Vice Versa, Chatley et al., in 2009, in a research on 64 cases with a 6-months followup period showed that the signal change indicative of a chronic constrictive lesion was a predictor of poor surgical results [10]. Naderi also found that age less than 60, normal preoperative cervical lordosis, and normal signal intensity (versus increased signal intensity on T2 MRI) within the spinal cord were associated with more favorable neurological postoperative improvements [20].

As we noted, we did not consider the surgical techniques and approaches as the effective variables and assumed that for each patient, a proper decompressive surgery with or without spinal stabilization (if indicated) has been carried out. In a relatively large systematic review carried out by Mummaneni et al., they used evidence-based medicine to judge against different surgical decompressive techniques commonly used in CSM patients [21]. Ultimately, the researchers concluded that these variable techniques had comparable outcome, and the surgical technique cannot be an important factor in determining the proper results, although they noted that laminectomy seemed to have a delayed worsening rate. This rule also applies to the patients with CSR, and decompression itself is still the main principle of treatment [22]. In our study, only six cases in Group B had laminectomy alone (without associated posterior instrumented fusion) and this small number could not have a great impact on the overall conclusions. 

In the study, we conducted that 88.2% of the CSR patients and 77.8% of the CSM patients had good or excellent results (patient satisfaction score 1 or 2), while 5.9% and 16.7%, respectively, showed postoperative deterioration (score 4). To compare the results, we looked at Kyung-Jin Song's and Radulovic's researches. Song et al. evaluated 76 patients with CSR who are treated by 1 or 2 level anterior cervical discectomy and fusion [23]. He categorized then into Group A (iliac crest bone graft alone) and Group B (iliac crest bone graft and plating). He reported 55% and 88.9% excellent or good results in Groups A and B, respectively. The other study (related to Radulović et al.) has been carried out on 57 surgically treated patients with CSM that showed good functional improvement irrelevant to the chosen surgical approach [18]. In this study, 75% of the cases showed postoperative improvement, while 21% remained unchanged and 4% deteriorated. In comparison with these two studies, our results in CSR and CSM's patients were similar.

The major limitations of our study are our retrospective method of research and diversity in surgical techniques. Obviously, a randomized control trial study will be able to offer stronger advices. In the future, it is suggested in order to decrease the confounding effect of the surgical type on the surgical outcome; it is better to compare the two groups with one similar surgical technique and approach. For example, a comparison between the results of surgical treatment of CSR and CSM in the patients treated with anterior cervical discectomy and fusion. 

In conclusion, surgery was associated with an improvement in the neck disability index in the both groups (*P* < 0.001). Regarding the improved NDI and patient's satisfaction score, functional results in both groups were similar and comparable. 

## Figures and Tables

**Table 1:** Demographic data of our treated patient.

	Number	Sex (M/F)	Mean age (range)	Followup (month)
Group A	68	20/48	48.2 ± 11.8 (30–73)	38.9 ± 10.4
Group B	72	50/22	55.7 ± 13.2 (31–80)	37.3 ± 8.2
*P* value	—	0.001^×^	0.14	0.49

^×^Statistically significant.

**Table 2:** Mean neck disability index and satisfaction rate in our patients.

	Preoperative NDI	Last visit NDI	Surgical effect (ΔNDI^×^)	Patient's satisfaction score
Group A	22.2 (6.1)^#^	9.1 (6.1)	12.6 (7.6)	1.6 (0.9)
Group B	27 (9.3)	11.4 (12)	15.8 (12.1)	1.7 (1.2)
*P* value	0.015∗	0.310	0.241	0.585

^×^Difference between preoperative and last indices.

^#^Standard deviation.

^*^Significant statistically.
